# [(*Z*)-*O*-Ethyl *N*-phenyl­thio­carbamato-κ*S*](tricyclo­hexyl­phosphine-κ*P*)gold(I): a monoclinic polymorph

**DOI:** 10.1107/S1600536810009086

**Published:** 2010-03-13

**Authors:** Primjira P. Tadbuppa, Edward R. T. Tiekink

**Affiliations:** a Department of Chemistry, National University of Singapore, Singapore 117543; b Department of Chemistry, University of Malaya, 50603 Kuala Lumpur, Malaysia

## Abstract

The title compound, [Au(C_9_H_10_NOS)(C_18_H_33_P)], represents a monoclinic polymorph to complement a previously reported triclinic (*P* 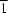) polymorph [Hall *et al.* (1993 ▶). *Aust. J. Chem.* **46**, 561–570 (unit-cell data only)]. The Au^I^ atom is coordinated within an *S*,*P*-donor set that defines a slightly distorted linear geometry [S—Au—P = = 175.43 (3)°], with the distortion due in part to a close intra­molecular Au⋯O contact [3.036 (2) Å]. In the crystal structure, mol­ecules are arranged into supra­molecular chains along the *b* axis mediated by C—H⋯π inter­actions.

## Related literature

For the structural systematics and luminescence properties of phosphinegold(I) carbonimidothio­ates, see: Ho *et al.* (2006 ▶); Ho & Tiekink (2007 ▶); Kuan *et al.* (2008 ▶). For the synthesis and for unit-cell data for the triclinic polymorph, see: Hall *et al.* (1993 ▶).
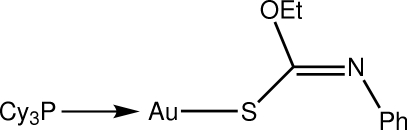


## Experimental

### 

#### Crystal data

[Au(C_9_H_10_NOS)(C_18_H_33_P)]
*M* *_r_* = 657.62
Monoclinic, 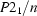

*a* = 16.1587 (7) Å
*b* = 9.1138 (4) Å
*c* = 18.7246 (9) Å
β = 90.448 (1)°
*V* = 2757.4 (2) Å^3^
*Z* = 4
Mo *K*α radiation
μ = 5.49 mm^−1^
*T* = 223 K
0.39 × 0.10 × 0.07 mm

#### Data collection

Bruker SMART CCD diffractometer
Absorption correction: multi-scan (*SADABS*; Bruker, 2000 ▶) *T*_min_ = 0.328, *T*_max_ = 1
18648 measured reflections
6309 independent reflections
5468 reflections with *I* > 2σ(*I*)
*R*_int_ = 0.045

#### Refinement

*R*[*F* ^2^ > 2σ(*F* ^2^)] = 0.025
*wR*(*F* ^2^) = 0.057
*S* = 1.01
6309 reflections
290 parameters
H-atom parameters constrained
Δρ_max_ = 0.69 e Å^−3^
Δρ_min_ = −1.03 e Å^−3^

### 

Data collection: *SMART* (Bruker, 2000 ▶); cell refinement: *SAINT* (Bruker, 2000 ▶); data reduction: *SAINT*; program(s) used to solve structure: *PATTY* in *DIRDIF92* (Beurskens *et al.*, 1992 ▶); program(s) used to refine structure: *SHELXL97* (Sheldrick, 2008 ▶); molecular graphics: *ORTEP-3* (Farrugia, 1997 ▶) and *DIAMOND* (Brandenburg, 2006 ▶); software used to prepare material for publication: *publCIF* (Westrip, 2010 ▶).

## Supplementary Material

Crystal structure: contains datablocks global, I. DOI: 10.1107/S1600536810009086/hg2657sup1.cif

Structure factors: contains datablocks I. DOI: 10.1107/S1600536810009086/hg2657Isup2.hkl

Additional supplementary materials:  crystallographic information; 3D view; checkCIF report

## Figures and Tables

**Table:** 

Au—P1	2.2687 (8)
Au—S1	2.3114 (8)

**Table:** 

P1—Au—S1	175.43 (3)

**Table 2:** Hydrogen-bond geometry (Å, °) *Cg* is the centroid of the C2–C7 ring.

*D*—H⋯*A*	*D*—H	H⋯*A*	*D*⋯*A*	*D*—H⋯*A*
C20—H20b⋯*Cg*^i^	0.98	2.98	3.689 (4)	130

Symmetry code: (i) 
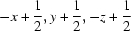
.

**Fig. 1.**
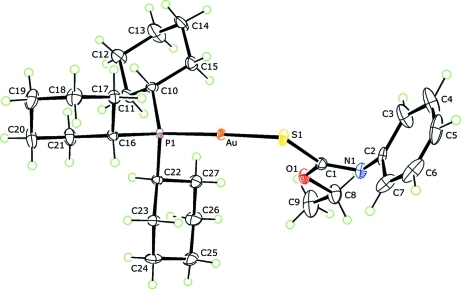
Molecular structure of (I) showing atom-labelling scheme and displacement ellipsoids at the 35% probability level.

**Fig. 2.**
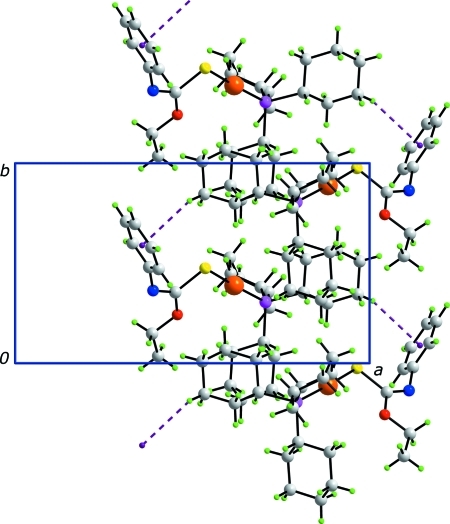
A view of a supramolecular chain in (I) aligned along the b axis with the C–H···π interactions shown as purple dashed lines. Colour code: Au, orange; S, yellow; P, pink; O, red; N, blue; C, grey; and H, green.

## supplementary crystallographic information

### Comment

Systematic structural studies of molecules with the general formula R_3_PAu[SC(OR')═NR''] for R, R' and R'' = alkyl and aryl (Ho *et al.* 2006; Ho & Tiekink, 2007; Kuan *et al.*, 2008), led to the investigation of the title compound, (I).

In keeping with expectation, the gold atom in (I) exists within an SP donor set defined by the phosphine-P and thiolate-S atoms, Table 1 and Fig. 1. Confirmation that the carbonimidothioate ligand is functioning as a thiolate is found in the magnitudes of the C1—S1 and C1═N1 distances of 1.749 (3) and 1.257 (4) Å, respectively. The coordination geometry is distorted from the ideal linear [S—Au—P = 175.43 (3) °] owing to the close approach of the O1 atom, 3.036 (2) Å. The most prominent interactions in the crystal structure are of the type C–H···π, Table 2, and these lead to the formation of supramolecular chains along the *b* axis, Fig. 2.

Unit cell data for a triclinic (P1) of (I) have been reported but no structural details were reported owing to the highly disordered nature of the molecule (Hall *et al.*, 1993).

### Experimental

Compound (I) was prepared following the standard literature procedure from the reaction of Cy_3_PAuCl and EtOC(═S)N(H)Ph in the presence of NaOH (Hall *et al.*, 1993). Crystals were obtained by the slow evaporation of a CH_2_Cl_2_/hexane (3/1) solution held at room temperature.

### Refinement

The H atoms were geometrically placed (C—H = 0.94-0.99 Å) and refined as riding with *U*_iso_(H) = 1.2-1.5*U*_eq_(C). The maximum and minimum residual electron density peaks of 0.69 and 1.03 e Å^-3^, respectively, were located 0.78 Å and 0.92 Å from the Au atom.

### Crystal data

**Table:**

[Au(C_9_H_10_NOS)(C_18_H_33_P)]	*F*(000) = 1320
*M**_r_* = 657.62	*D*_x_ = 1.584 Mg m^−^^3^
Monoclinic, *P*2_1_/*n*	Mo *K*α radiation, λ = 0.71069 Å
Hall symbol: -P 2yn	Cell parameters from 6662 reflections
*a* = 16.1587 (7) Å	θ = 2.5–29.9°
*b* = 9.1138 (4) Å	µ = 5.49 mm^−^^1^
*c* = 18.7246 (9) Å	*T* = 223 K
β = 90.448 (1)°	Prism, colourless
*V* = 2757.4 (2) Å^3^	0.39 × 0.10 × 0.07 mm
*Z* = 4	

### Data collection

**Table:**

Bruker SMART CCD diffractometer	6309 independent reflections
Radiation source: fine-focus sealed tube	5468 reflections with *I* > 2σ(*I*)
graphite	*R*_int_ = 0.045
ω scans	θ_max_ = 27.5°, θ_min_ = 1.7°
Absorption correction: multi-scan (*SADABS*; Bruker, 2000)	*h* = −18→20
*T*_min_ = 0.328, *T*_max_ = 1	*k* = −9→11
18648 measured reflections	*l* = −24→23

### Refinement

**Table:**

Refinement on *F*^2^	Primary atom site location: structure-invariant direct methods
Least-squares matrix: full	Secondary atom site location: difference Fourier map
*R*[*F*^2^ > 2σ(*F*^2^)] = 0.025	Hydrogen site location: inferred from neighbouring sites
*wR*(*F*^2^) = 0.057	H-atom parameters constrained
*S* = 1.01	*w* = 1/[σ^2^(*F*_o_^2^) + (0.0174*P*)^2^] where *P* = (*F*_o_^2^ + 2*F*_c_^2^)/3
6309 reflections	(Δ/σ)_max_ = 0.001
290 parameters	Δρ_max_ = 0.69 e Å^−^^3^
0 restraints	Δρ_min_ = −1.03 e Å^−^^3^

### Special details

**Table:**

Geometry. All s.u.'s (except the s.u. in the dihedral angle between two l.s. planes) are estimated using the full covariance matrix. The cell s.u.'s are taken into account individually in the estimation of s.u.'s in distances, angles and torsion angles; correlations between s.u.'s in cell parameters are only used when they are defined by crystal symmetry. An approximate (isotropic) treatment of cell s.u.'s is used for estimating s.u.'s involving l.s. planes.
Refinement. Refinement of *F*^2^ against ALL reflections. The weighted *R*-factor wR and goodness of fit *S* are based on *F*^2^, conventional *R*-factors *R* are based on *F*, with *F* set to zero for negative *F*^2^. The threshold expression of *F*^2^ > 2σ(*F*^2^) is used only for calculating *R*-factors(gt) etc. and is not relevant to the choice of reflections for refinement. *R*-factors based on *F*^2^ are statistically about twice as large as those based on *F*, and *R*- factors based on ALL data will be even larger.

### Fractional atomic coordinates and isotropic or equivalent isotropic displacement parameters (Å^2^)

**Table:**

	*x*	*y*	*z*	*U*_iso_*/*U*_eq_	
Au	0.117624 (6)	0.111622 (12)	0.183207 (6)	0.01601 (4)	
S1	0.03607 (5)	0.02561 (9)	0.27503 (5)	0.02352 (18)	
P1	0.20646 (4)	0.19446 (8)	0.09908 (4)	0.01485 (16)	
O1	−0.04247 (12)	0.2578 (2)	0.22844 (12)	0.0247 (5)	
N1	−0.11098 (15)	0.1371 (3)	0.31544 (15)	0.0275 (7)	
C1	−0.04832 (18)	0.1457 (4)	0.27632 (17)	0.0202 (7)	
C2	−0.12197 (18)	0.0201 (4)	0.36357 (19)	0.0271 (8)	
C3	−0.1686 (2)	−0.0999 (5)	0.3430 (3)	0.0497 (12)	
H3	−0.1876	−0.1085	0.2956	0.060*	
C4	−0.1874 (2)	−0.2076 (6)	0.3926 (3)	0.0658 (16)	
H4	−0.2181	−0.2900	0.3782	0.079*	
C5	−0.1621 (3)	−0.1962 (6)	0.4617 (3)	0.0577 (14)	
H5	−0.1765	−0.2689	0.4950	0.069*	
C6	−0.1157 (3)	−0.0787 (5)	0.4826 (2)	0.0533 (13)	
H6	−0.0974	−0.0709	0.5302	0.064*	
C7	−0.0953 (2)	0.0294 (5)	0.4335 (2)	0.0424 (10)	
H7	−0.0630	0.1099	0.4482	0.051*	
C8	−0.1138 (2)	0.3525 (4)	0.2245 (2)	0.0387 (10)	
H8A	−0.1228	0.4006	0.2706	0.046*	
H8B	−0.1633	0.2957	0.2121	0.046*	
C9	−0.0966 (3)	0.4645 (5)	0.1680 (3)	0.0651 (16)	
H9A	−0.0493	0.5234	0.1821	0.098*	
H9B	−0.1446	0.5274	0.1619	0.098*	
H9C	−0.0849	0.4153	0.1232	0.098*	
C10	0.1927 (2)	0.1200 (3)	0.00840 (17)	0.0206 (7)	
H10	0.2318	0.0366	0.0056	0.025*	
C11	0.2174 (2)	0.2222 (4)	−0.05252 (17)	0.0252 (7)	
H11A	0.2737	0.2587	−0.0439	0.030*	
H11B	0.1800	0.3068	−0.0537	0.030*	
C12	0.2141 (2)	0.1444 (4)	−0.12399 (19)	0.0378 (9)	
H12A	0.2256	0.2151	−0.1621	0.045*	
H12B	0.2570	0.0686	−0.1252	0.045*	
C13	0.1312 (3)	0.0750 (5)	−0.1373 (2)	0.0545 (13)	
H13A	0.1331	0.0193	−0.1820	0.065*	
H13B	0.0895	0.1524	−0.1430	0.065*	
C14	0.1053 (2)	−0.0270 (4)	−0.0771 (2)	0.0386 (10)	
H14A	0.0491	−0.0631	−0.0864	0.046*	
H14B	0.1426	−0.1118	−0.0754	0.046*	
C15	0.1079 (2)	0.0531 (4)	−0.00506 (19)	0.0301 (8)	
H15A	0.0659	0.1307	−0.0048	0.036*	
H15B	0.0950	−0.0161	0.0333	0.036*	
C16	0.31353 (18)	0.1463 (3)	0.12613 (17)	0.0186 (7)	
H16	0.3237	0.1925	0.1732	0.022*	
C17	0.32012 (19)	−0.0192 (3)	0.13699 (18)	0.0239 (7)	
H17A	0.3066	−0.0691	0.0920	0.029*	
H17B	0.2796	−0.0501	0.1726	0.029*	
C18	0.4071 (2)	−0.0660 (4)	0.1619 (2)	0.0357 (9)	
H18A	0.4179	−0.0275	0.2099	0.043*	
H18B	0.4101	−0.1733	0.1640	0.043*	
C19	0.4719 (2)	−0.0092 (4)	0.1113 (2)	0.0407 (10)	
H19A	0.5270	−0.0353	0.1296	0.049*	
H19B	0.4646	−0.0558	0.0646	0.049*	
C20	0.46621 (19)	0.1550 (4)	0.1030 (2)	0.0407 (10)	
H20A	0.4779	0.2021	0.1491	0.049*	
H20B	0.5080	0.1883	0.0690	0.049*	
C21	0.38063 (19)	0.2009 (4)	0.0767 (2)	0.0309 (8)	
H21A	0.3780	0.3082	0.0737	0.037*	
H21B	0.3711	0.1613	0.0287	0.037*	
C22	0.20142 (18)	0.3951 (3)	0.09267 (17)	0.0173 (6)	
H22	0.2379	0.4275	0.0535	0.021*	
C23	0.2312 (2)	0.4662 (3)	0.16227 (17)	0.0226 (7)	
H23A	0.2890	0.4386	0.1713	0.027*	
H23B	0.1979	0.4297	0.2020	0.027*	
C24	0.2244 (2)	0.6331 (3)	0.1587 (2)	0.0300 (8)	
H24A	0.2413	0.6749	0.2048	0.036*	
H24B	0.2622	0.6703	0.1223	0.036*	
C25	0.1374 (2)	0.6827 (4)	0.1411 (2)	0.0346 (9)	
H25A	0.1363	0.7897	0.1361	0.041*	
H25B	0.1004	0.6557	0.1802	0.041*	
C26	0.1068 (2)	0.6113 (3)	0.0713 (2)	0.0356 (9)	
H26A	0.0492	0.6400	0.0622	0.043*	
H26B	0.1403	0.6465	0.0314	0.043*	
C27	0.11259 (19)	0.4447 (4)	0.0756 (2)	0.0288 (8)	
H27A	0.0947	0.4020	0.0300	0.035*	
H27B	0.0754	0.4089	0.1128	0.035*	

### Atomic displacement parameters (Å^2^)

**Table:**

	*U*^11^	*U*^22^	*U*^33^	*U*^12^	*U*^13^	*U*^23^
Au	0.01606 (7)	0.01665 (7)	0.01538 (7)	−0.00120 (5)	0.00363 (4)	0.00123 (5)
S1	0.0197 (4)	0.0271 (4)	0.0238 (4)	0.0031 (3)	0.0081 (3)	0.0092 (4)
P1	0.0161 (4)	0.0142 (4)	0.0143 (4)	−0.0029 (3)	0.0035 (3)	0.0005 (3)
O1	0.0172 (11)	0.0253 (12)	0.0317 (14)	0.0033 (10)	0.0014 (9)	0.0071 (11)
N1	0.0210 (14)	0.0333 (18)	0.0284 (17)	0.0005 (12)	0.0071 (12)	0.0008 (13)
C1	0.0176 (15)	0.0240 (17)	0.0188 (17)	−0.0008 (13)	−0.0011 (12)	−0.0015 (13)
C2	0.0142 (15)	0.038 (2)	0.029 (2)	0.0067 (15)	0.0108 (13)	0.0069 (16)
C3	0.039 (2)	0.066 (3)	0.044 (3)	−0.024 (2)	−0.0044 (19)	0.021 (2)
C4	0.037 (2)	0.081 (4)	0.079 (4)	−0.026 (2)	−0.003 (2)	0.044 (3)
C5	0.039 (2)	0.081 (4)	0.053 (3)	0.017 (2)	0.028 (2)	0.040 (3)
C6	0.063 (3)	0.069 (3)	0.028 (2)	0.039 (3)	0.010 (2)	0.005 (2)
C7	0.049 (2)	0.046 (3)	0.032 (2)	0.021 (2)	0.0047 (18)	−0.0040 (19)
C8	0.0250 (18)	0.035 (2)	0.056 (3)	0.0094 (17)	0.0031 (17)	0.015 (2)
C9	0.038 (2)	0.059 (3)	0.098 (4)	0.019 (2)	0.013 (2)	0.045 (3)
C10	0.0273 (17)	0.0185 (16)	0.0161 (17)	−0.0043 (13)	0.0036 (13)	−0.0021 (13)
C11	0.0290 (17)	0.0302 (19)	0.0165 (17)	−0.0132 (15)	0.0018 (13)	0.0030 (14)
C12	0.048 (2)	0.049 (2)	0.0159 (19)	−0.020 (2)	0.0082 (16)	−0.0027 (17)
C13	0.060 (3)	0.086 (3)	0.018 (2)	−0.036 (3)	−0.0020 (19)	−0.009 (2)
C14	0.035 (2)	0.050 (2)	0.031 (2)	−0.0275 (19)	0.0014 (16)	−0.0104 (19)
C15	0.0273 (18)	0.040 (2)	0.0228 (19)	−0.0099 (17)	0.0040 (14)	−0.0038 (17)
C16	0.0201 (15)	0.0162 (16)	0.0196 (17)	−0.0033 (13)	0.0024 (12)	0.0012 (13)
C17	0.0241 (16)	0.0191 (17)	0.0284 (19)	0.0005 (14)	0.0006 (14)	0.0052 (14)
C18	0.033 (2)	0.032 (2)	0.042 (2)	0.0083 (17)	0.0012 (17)	0.0108 (18)
C19	0.0258 (19)	0.044 (2)	0.052 (3)	0.0144 (17)	0.0063 (17)	0.009 (2)
C20	0.0159 (17)	0.046 (2)	0.060 (3)	−0.0023 (17)	0.0051 (17)	0.017 (2)
C21	0.0236 (17)	0.0274 (19)	0.042 (2)	−0.0009 (15)	0.0054 (15)	0.0130 (17)
C22	0.0203 (15)	0.0151 (15)	0.0164 (16)	−0.0017 (12)	0.0002 (12)	0.0005 (12)
C23	0.0288 (17)	0.0185 (17)	0.0204 (18)	−0.0021 (14)	0.0002 (13)	−0.0027 (14)
C24	0.048 (2)	0.0155 (17)	0.027 (2)	−0.0046 (16)	0.0011 (16)	−0.0041 (14)
C25	0.042 (2)	0.0171 (18)	0.044 (2)	0.0057 (16)	0.0068 (18)	−0.0020 (17)
C26	0.039 (2)	0.0241 (19)	0.043 (2)	0.0098 (16)	−0.0061 (18)	0.0016 (17)
C27	0.0243 (17)	0.0246 (18)	0.037 (2)	0.0051 (15)	−0.0049 (15)	−0.0035 (16)

### Geometric parameters (Å, °)

**Table:**

Au—P1	2.2687 (8)	C14—C15	1.534 (5)
Au—S1	2.3114 (8)	C14—H14A	0.9800
S1—C1	1.749 (3)	C14—H14B	0.9800
P1—C22	1.834 (3)	C15—H15A	0.9800
P1—C16	1.852 (3)	C15—H15B	0.9800
P1—C10	1.840 (3)	C16—C21	1.515 (4)
O1—C1	1.363 (4)	C16—C17	1.526 (4)
O1—C8	1.441 (4)	C16—H16	0.9900
N1—C1	1.257 (4)	C17—C18	1.538 (4)
N1—C2	1.408 (4)	C17—H17A	0.9800
C2—C3	1.382 (5)	C17—H17B	0.9800
C2—C7	1.378 (5)	C18—C19	1.508 (5)
C3—C4	1.387 (5)	C18—H18A	0.9800
C3—H3	0.9400	C18—H18B	0.9800
C4—C5	1.358 (7)	C19—C20	1.507 (5)
C4—H4	0.9400	C19—H19A	0.9800
C5—C6	1.362 (7)	C19—H19B	0.9800
C5—H5	0.9400	C20—C21	1.523 (4)
C6—C7	1.389 (6)	C20—H20A	0.9800
C6—H6	0.9400	C20—H20B	0.9800
C7—H7	0.9400	C21—H21A	0.9800
C8—C9	1.498 (5)	C21—H21B	0.9800
C8—H8A	0.9800	C22—C23	1.530 (4)
C8—H8B	0.9800	C22—C27	1.536 (4)
C9—H9A	0.9700	C22—H22	0.9900
C9—H9B	0.9700	C23—C24	1.527 (4)
C9—H9C	0.9700	C23—H23A	0.9800
C10—C15	1.520 (4)	C23—H23B	0.9800
C10—C11	1.528 (4)	C24—C25	1.511 (5)
C10—H10	0.9900	C24—H24A	0.9800
C11—C12	1.515 (5)	C24—H24B	0.9800
C11—H11A	0.9800	C25—C26	1.538 (5)
C11—H11B	0.9800	C25—H25A	0.9800
C12—C13	1.500 (5)	C25—H25B	0.9800
C12—H12A	0.9800	C26—C27	1.523 (4)
C12—H12B	0.9800	C26—H26A	0.9800
C13—C14	1.523 (5)	C26—H26B	0.9800
C13—H13A	0.9800	C27—H27A	0.9800
C13—H13B	0.9800	C27—H27B	0.9800
			
P1—Au—S1	175.43 (3)	C10—C15—H15B	109.5
C1—S1—Au	104.30 (11)	C14—C15—H15B	109.5
C22—P1—C16	107.15 (14)	H15A—C15—H15B	108.1
C22—P1—C10	107.59 (14)	C21—C16—C17	110.9 (3)
C16—P1—C10	105.70 (15)	C21—C16—P1	115.3 (2)
C22—P1—Au	110.44 (11)	C17—C16—P1	109.6 (2)
C16—P1—Au	109.04 (10)	C21—C16—H16	106.9
C10—P1—Au	116.46 (10)	C17—C16—H16	106.9
C1—O1—C8	115.0 (2)	P1—C16—H16	106.9
C1—N1—C2	121.8 (3)	C16—C17—C18	112.1 (3)
N1—C1—O1	119.4 (3)	C16—C17—H17A	109.2
N1—C1—S1	126.9 (3)	C18—C17—H17A	109.2
O1—C1—S1	113.7 (2)	C16—C17—H17B	109.2
C3—C2—C7	118.6 (4)	C18—C17—H17B	109.2
C3—C2—N1	119.5 (3)	H17A—C17—H17B	107.9
C7—C2—N1	121.4 (4)	C19—C18—C17	110.7 (3)
C2—C3—C4	119.7 (4)	C19—C18—H18A	109.5
C2—C3—H3	120.2	C17—C18—H18A	109.5
C4—C3—H3	120.2	C19—C18—H18B	109.5
C5—C4—C3	121.3 (5)	C17—C18—H18B	109.5
C5—C4—H4	119.4	H18A—C18—H18B	108.1
C3—C4—H4	119.4	C20—C19—C18	111.3 (3)
C4—C5—C6	119.6 (4)	C20—C19—H19A	109.4
C4—C5—H5	120.2	C18—C19—H19A	109.4
C6—C5—H5	120.2	C20—C19—H19B	109.4
C7—C6—C5	120.1 (4)	C18—C19—H19B	109.4
C7—C6—H6	120.0	H19A—C19—H19B	108.0
C5—C6—H6	120.0	C21—C20—C19	111.2 (3)
C2—C7—C6	120.7 (4)	C21—C20—H20A	109.4
C2—C7—H7	119.6	C19—C20—H20A	109.4
C6—C7—H7	119.6	C21—C20—H20B	109.4
O1—C8—C9	107.0 (3)	C19—C20—H20B	109.4
O1—C8—H8A	110.3	H20A—C20—H20B	108.0
C9—C8—H8A	110.3	C16—C21—C20	111.4 (3)
O1—C8—H8B	110.3	C16—C21—H21A	109.4
C9—C8—H8B	110.3	C20—C21—H21A	109.4
H8A—C8—H8B	108.6	C16—C21—H21B	109.4
C8—C9—H9A	109.5	C20—C21—H21B	109.4
C8—C9—H9B	109.5	H21A—C21—H21B	108.0
H9A—C9—H9B	109.5	C23—C22—C27	109.9 (3)
C8—C9—H9C	109.5	C23—C22—P1	110.7 (2)
H9A—C9—H9C	109.5	C27—C22—P1	110.4 (2)
H9B—C9—H9C	109.5	C23—C22—H22	108.6
C15—C10—C11	111.2 (3)	C27—C22—H22	108.6
C15—C10—P1	113.8 (2)	P1—C22—H22	108.6
C11—C10—P1	115.7 (2)	C22—C23—C24	111.3 (3)
C15—C10—H10	105.0	C22—C23—H23A	109.4
C11—C10—H10	105.0	C24—C23—H23A	109.4
P1—C10—H10	105.0	C22—C23—H23B	109.4
C10—C11—C12	111.5 (3)	C24—C23—H23B	109.4
C10—C11—H11A	109.3	H23A—C23—H23B	108.0
C12—C11—H11A	109.3	C25—C24—C23	112.0 (3)
C10—C11—H11B	109.3	C25—C24—H24A	109.2
C12—C11—H11B	109.3	C23—C24—H24A	109.2
H11A—C11—H11B	108.0	C25—C24—H24B	109.2
C13—C12—C11	111.7 (3)	C23—C24—H24B	109.2
C13—C12—H12A	109.3	H24A—C24—H24B	107.9
C11—C12—H12A	109.3	C24—C25—C26	110.6 (3)
C13—C12—H12B	109.3	C24—C25—H25A	109.5
C11—C12—H12B	109.3	C26—C25—H25A	109.5
H12A—C12—H12B	107.9	C24—C25—H25B	109.5
C12—C13—C14	112.6 (3)	C26—C25—H25B	109.5
C12—C13—H13A	109.1	H25A—C25—H25B	108.1
C14—C13—H13A	109.1	C27—C26—C25	110.9 (3)
C12—C13—H13B	109.1	C27—C26—H26A	109.5
C14—C13—H13B	109.1	C25—C26—H26A	109.5
H13A—C13—H13B	107.8	C27—C26—H26B	109.5
C15—C14—C13	110.8 (3)	C25—C26—H26B	109.5
C15—C14—H14A	109.5	H26A—C26—H26B	108.0
C13—C14—H14A	109.5	C26—C27—C22	111.2 (3)
C15—C14—H14B	109.5	C26—C27—H27A	109.4
C13—C14—H14B	109.5	C22—C27—H27A	109.4
H14A—C14—H14B	108.1	C26—C27—H27B	109.4
C10—C15—C14	110.8 (3)	C22—C27—H27B	109.4
C10—C15—H15A	109.5	H27A—C27—H27B	108.0
C14—C15—H15A	109.5		
			
P1—Au—S1—C1	132.9 (4)	C11—C10—C15—C14	55.9 (4)
S1—Au—P1—C22	−100.4 (4)	P1—C10—C15—C14	−171.4 (3)
S1—Au—P1—C16	17.1 (4)	C13—C14—C15—C10	−55.1 (4)
S1—Au—P1—C10	136.6 (4)	C22—P1—C16—C21	−57.7 (3)
C2—N1—C1—O1	177.1 (3)	C10—P1—C16—C21	56.8 (3)
C2—N1—C1—S1	−2.4 (5)	Au—P1—C16—C21	−177.2 (2)
C8—O1—C1—N1	−3.9 (4)	C22—P1—C16—C17	176.4 (2)
C8—O1—C1—S1	175.6 (2)	C10—P1—C16—C17	−69.1 (2)
Au—S1—C1—N1	176.1 (3)	Au—P1—C16—C17	56.9 (2)
Au—S1—C1—O1	−3.4 (2)	C21—C16—C17—C18	53.4 (4)
C1—N1—C2—C3	−95.3 (4)	P1—C16—C17—C18	−178.2 (2)
C1—N1—C2—C7	92.2 (4)	C16—C17—C18—C19	−54.1 (4)
C7—C2—C3—C4	−0.1 (6)	C17—C18—C19—C20	55.9 (4)
N1—C2—C3—C4	−172.8 (4)	C18—C19—C20—C21	−57.5 (5)
C2—C3—C4—C5	1.3 (7)	C17—C16—C21—C20	−54.4 (4)
C3—C4—C5—C6	−1.7 (7)	P1—C16—C21—C20	−179.6 (3)
C4—C5—C6—C7	0.8 (6)	C19—C20—C21—C16	56.7 (4)
C3—C2—C7—C6	−0.7 (5)	C16—P1—C22—C23	−54.1 (3)
N1—C2—C7—C6	171.9 (3)	C10—P1—C22—C23	−167.4 (2)
C5—C6—C7—C2	0.4 (6)	Au—P1—C22—C23	64.5 (2)
C1—O1—C8—C9	−178.3 (3)	C16—P1—C22—C27	−175.9 (2)
C22—P1—C10—C15	−105.1 (2)	C10—P1—C22—C27	70.8 (3)
C16—P1—C10—C15	140.6 (2)	Au—P1—C22—C27	−57.3 (2)
Au—P1—C10—C15	19.4 (3)	C27—C22—C23—C24	−55.8 (3)
C22—P1—C10—C11	25.4 (3)	P1—C22—C23—C24	−177.9 (2)
C16—P1—C10—C11	−88.8 (3)	C22—C23—C24—C25	56.1 (4)
Au—P1—C10—C11	150.0 (2)	C23—C24—C25—C26	−55.4 (4)
C15—C10—C11—C12	−55.4 (4)	C24—C25—C26—C27	55.8 (4)
P1—C10—C11—C12	172.8 (2)	C25—C26—C27—C22	−57.0 (4)
C10—C11—C12—C13	54.1 (4)	C23—C22—C27—C26	56.7 (4)
C11—C12—C13—C14	−54.2 (5)	P1—C22—C27—C26	179.1 (3)
C12—C13—C14—C15	54.5 (5)		

### Hydrogen-bond geometry (Å, °)

**Table:**

Cg is the centroid of the C2–C7 ring.

**Table:**

*D*—H···*A*	*D*—H	H···*A*	*D*···*A*	*D*—H···*A*
C20—H20b···Cg^i^	0.98	2.98	3.689 (4)	130

Symmetry codes: (i) −*x*+1/2, *y*+1/2, −*z*+1/2.

## References

[bb1] Beurskens, P. T., Admiraal, G., Beurskens, G., Bosman, W. P., Garcia-Granda, S., Gould, R. O., Smits, J. M. M. & Smykalla, C. (1992). *The *DIRDIF* Program System* Technical Report. Crystallography Laboratory, University of Nijmegen, The Netherlands.

[bb2] Brandenburg, K. (2006). *DIAMOND* Crystal Impact GbR, Bonn, Germany.

[bb3] Bruker (2000). *SMART*, *SAINT* and *SADABS* Bruker AXS Inc., Madison, Wisconsin, USA.

[bb4] Farrugia, L. J. (1997). *J. Appl. Cryst.* **30**, 565.

[bb5] Hall, V. J., Siasios, G. & Tiekink, E. R. T. (1993). *Aust. J. Chem.* **46**, 561–570.

[bb6] Ho, S. Y., Cheng, E. C.-C., Tiekink, E. R. T. & Yam, V. W.-W. (2006). *Inorg. Chem.* **45**, 8165–8174. 10.1021/ic0608243 16999414

[bb7] Ho, S. Y. & Tiekink, E. R. T. (2007). *CrystEngComm*, **9**, 368–378.

[bb8] Kuan, F. S., Ho, S. Y., Tadbuppa, P. P. & Tiekink, E. R. T. (2008). *CrystEngComm*, **10**, 548–564.

[bb9] Sheldrick, G. M. (2008). *Acta Cryst.* A**64**, 112–122. 10.1107/S0108767307043930 18156677

[bb10] Westrip, S. P. (2010). *publCIF* In preparation.

